# Cell-Free Hemoglobin in Acute Kidney Injury after Lung Transplantation and Experimental Renal Ischemia/Reperfusion

**DOI:** 10.3390/ijms232113272

**Published:** 2022-10-31

**Authors:** Robert Greite, Li Wang, Lukas Gohlke, Sebastian Schott, Kirill Kreimann, Julian Doricic, Andreas Leffler, Igor Tudorache, Jawad Salman, Ruslan Natanov, Fabio Ius, Christine Fegbeutel, Axel Haverich, Ralf Lichtinghagen, Rongjun Chen, Song Rong, Hermann Haller, Vijith Vijayan, Magnus Gram, Irina Scheffner, Faikah Gueler, Wilfried Gwinner, Stephan Immenschuh

**Affiliations:** 1 Department of Nephrology and Hypertension, Hannover Medical School, 30625 Hannover, Germany; 2 Anaesthesiology, Hannover Medical School, 30625 Hannover, Germany; 3 Cardiac Surgery, University of Dusseldorf, 40225 Dusseldorf, Germany; 4 Cardiac Surgery, Hannover Medical School, 30625 Hannover, Germany; 5 German Center for Lung Research (DZL), 35392 Giessen, Germany; 6 Clinical Chemistry, Hannover Medical School, 30625 Hannover, Germany; 7 Pediatrics, Stanford University, Stanford, CA 94305, USA; 8 Department of Pediatrics, Clinical Sciences Lund, Lund University, 22220 Lund, Sweden; 9 Institute of Transfusion Medicine and Transplant Engineering, Hannover Medical School, 30625 Hannover, Germany

**Keywords:** acute kidney injury, free hemoglobin, hemolysis, lung transplantation

## Abstract

Cell-free hemoglobin (CFH), a pro-oxidant and cytotoxic compound that is released in hemolysis, has been associated with nephrotoxicity. Lung transplantation (LuTx) is a clinical condition with a high incidence of acute kidney injury (AKI). In this study, we investigated the plasma levels of CFH and haptoglobin, a CFH-binding serum protein, in prospectively enrolled LuTx patients (*n* = 20) with and without AKI. LuTx patients with postoperative AKI had higher CFH plasma levels at the end of surgery compared with no-AKI patients, and CFH correlated with serum creatinine at 48 h. Moreover, CFH levels inversely correlated with haptoglobin levels, which were significantly reduced at the end of surgery in LuTx patients with AKI. Because multiple other factors can contribute to AKI development in the complex clinical setting of LuTx, we next investigated the role of exogenous CFH administration in a mouse model of mild bilateral renal ischemia reperfusion injury (IRI). Exogenous administration of CFH after reperfusion caused overt AKI with creatinine increase, tubular injury, and enhanced markers of renal inflammation compared with vehicle-treated animals. In conclusion, CFH is a possible factor contributing to postoperative AKI after LuTx and promotes AKI in an experimental model of mild transient renal ischemia. Targeting CFH might be a therapeutic option to prevent AKI after LuTx.

## 1. Introduction

Acute kidney injury (AKI) frequently complicates lung transplantation (LuTx) [1,2,3] and is associated with increased risk of death [4,5,6]. The pathogenesis of AKI after LuTx is multifactorial, including systemic inflammation and nephrotoxic side effects of pharmacological immunosuppression. Moreover, transient kidney hypo-perfusion with subsequent renal ischemia reperfusion injury (IRI) and the need for extracorporeal membrane oxygenation (ECMO) have been associated with AKI after LuTx [7,8]. In particular, the shear-stress-induced red blood cell (RBC) damage associated with ECMO support and transfusion of RBC units leads to hemolysis with high levels of cell-free hemoglobin (CFH) in this condition [9,10]. CFH can be toxic via interactions with nitric oxide and its pro-oxidant effects. Moreover, CFH can release redox reactive free heme that may damage lipids, proteins, and DNA. In addition, CFH can translocate from the intravascular to the extravascular space and may cause detrimental effects in the parenchyma of various organs such as the kidney [11]. The nephrotoxic effects of CFH have been demonstrated in several in vitro [12,13] and in vivo models of transfusion [14], sickle cell disease [15], and sepsis [16], but the role of CFH in the development of AKI after LuTx is largely unknown. To investigate the role of CFH for AKI in LuTx, we chose two approaches in this study. First, we examined the association of plasma CFH levels with AKI in a cohort of LuTx patients. Second, because the clinical setting of LuTx is complex and multiple factors can contribute to AKI in this condition, a mouse model of mild experimental renal IRI was applied to determine specific effects of CFH in transient renal ischemia. The findings suggest that CFH is a critical factor for the pathogenesis of AKI in LuTx.

## 2. Results

### 2.1. Patient Characteristics and Pre-, Peri-, and Postoperative Factors

The clinical relevance of CFH in AKI was assessed in a cohort of LuTx patients. The patient characteristics and pre-, peri-, and postoperative factors are detailed in Table 1. The need for intraoperative ECMO was not different between the groups (*n* = 2 in the no-AKI and *n* = 3 patients in the AKI-group, Table 1). The other patients underwent surgery without extracorporeal circulation. The ischemia times of the first and second lung were significantly higher in patients that developed postoperative AKI. The transfusion of blood products was comparable between AKI- and no-AKI patients.

### 2.2. CFH and Haptoglobin Levels in LuTx Patients

**Figure 1:**
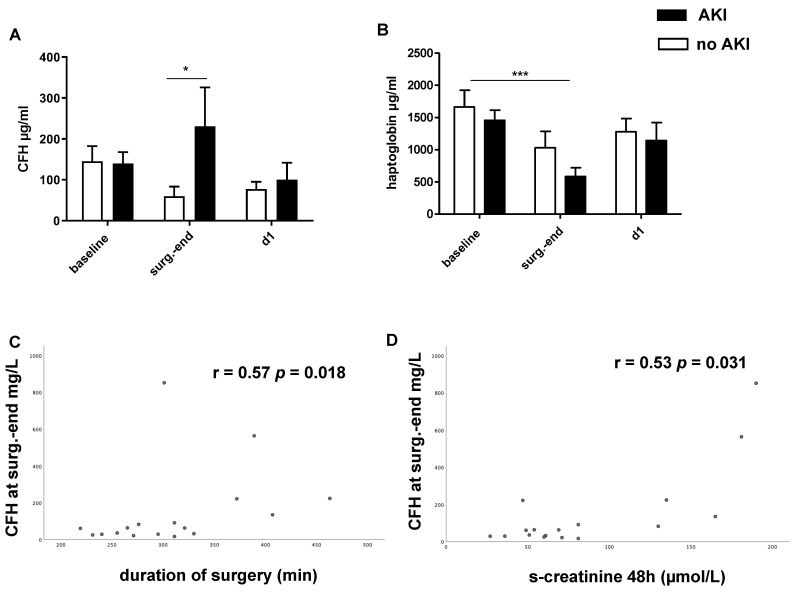
Plasma-free hemoglobin (CFH, (**A**)) and haptoglobin levels (**B**) in lung transplant (LuTx) patients with AKI (black bar) or without AKI (white bar). CFH was significantly higher at the end of surgery in LuTx patients with subsequent AKI (**A**). In parallel, a marked decline in plasma haptoglobin levels was found at the end of LuTx (**B**). CFH levels at the end of surgery (surg.-end) significantly correlated with duration of surgery (**C**) and serum creatinine 48 h after LuTx (**D**). Spearman’s r and *p*-values are shown. * *p* < 0.05, *** *p* < 0.001.

Baseline CFH levels were immediately obtained after anesthesia induction. A moderate increase in CFH levels was observed at the end of surgery in patients with postoperative AKI (Figure 1A). CFH levels reached baseline values by day 1. The levels of the CFH-binding protein haptoglobin were significantly lower compared with baseline in the AKI group at the end of surgery (Figure 1B). The CFH levels at the end of surgery correlated with duration of surgery (r = 0.57; *p* = 0.018) (Figure 1C) and serum creatinine 48 h after LuTx (r = 0.53; *p* = 0.031) (Figure 1D). As a reference value for CFH, <100 µg/mL [17,18,19,20] was considered for healthy patients. A moderate correlation between CFH levels and ischemia times of the first (r = 0.5; *p* = 0.06) and second donor lung (r = 0.5; *p* = 0.07), which did not reach statistical significance, was observed.

### 2.3. AKI and Distant Organ Injury in a Mouse Model of Mild Renal IRI and Exogenous CFH Administration

The pathophysiological events that may cause AKI in LuTx are highly complex and include multiple factors. To investigate the specific role of exogenous CFH in transient renal ischemia, we applied a mouse model of short-term bilateral renal IRI (15 min).

**Figure 2:**
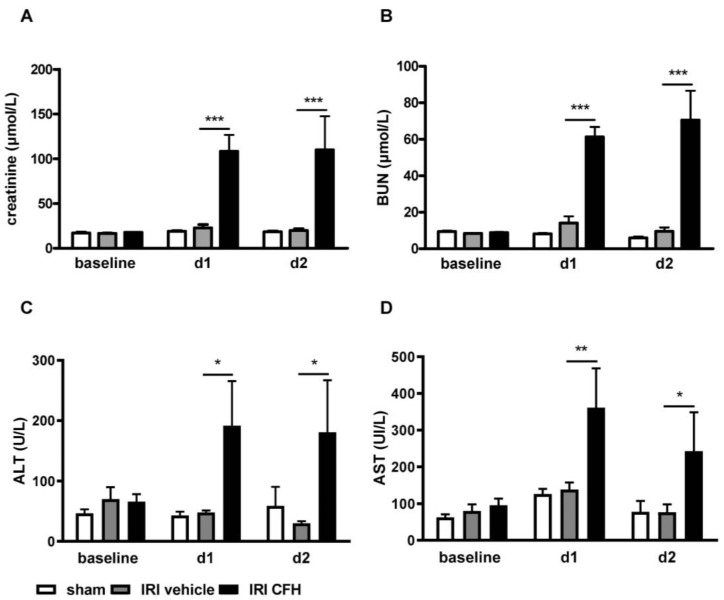
Serum creatinine (**A**), blood urea nitrogen (BUN) (**B**) and the liver enzymes ALT (**C**) and AST (**D**) in mice before (baseline) and at days 1 (d1) and 2 (d2) after sham surgery (white bar) and bilateral renal ischemia reperfusion injury (IRI) for 15 min with vehicle (grey bar) or cell free-hemoglobin (CFH, black bar) treatment. Bilateral renal IRI (with vehicle) did not cause acute kidney injury (AKI) in this model as evidenced by normal serum creatinine ((**A**), grey bar) and BUN ((**B**), grey bar) levels. However, CFH administration in this model of subclinical renal IRI caused overt AKI with marked increases in serum creatinine ((**A**), black bar) and BUN ((**B**), black bar) levels in this subclinical IRI model. Moreover, signs of distant organ injury such as elevation of the liver enzymes ALT ((**C**), black bar) and AST ((**D**), black bar) were observed after renal IRI with CFH administration. * *p* < 0.05, ** *p* < 0.01, *** *p* < 0.001.

The exogenous administration of CFH after reperfusion, but not vehicle, caused a marked increase in serum creatinine and blood urea nitrogen (BUN) levels after 24 and 48 h (Figure 2A,B). The liver enzymes alanine-aminotransferase (ALT) and aspartate-aminotransferase (AST) were also significantly elevated in the plasma of IRI mice treated with CFH (Figure 2C,D), indicating that CFH may induce distant organ injury in subclinical renal IRI. IRI-vehicle mice and sham-operated mice had normal ALT and AST levels (Figure 2C,D).

**Figure 3:**
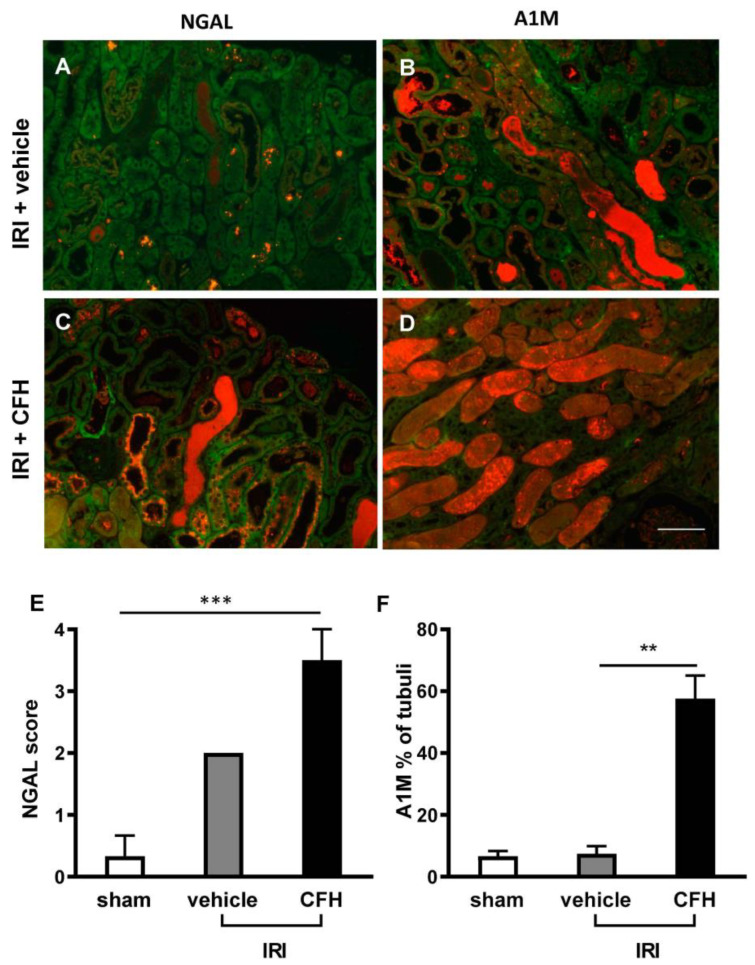
Kidney immunofluorescence staining for the renal injury marker NGAL (**A**,**C**) and the tubular transport marker A1M (**B**,**D**) in mice 48 h after IRI with or without CFH administration. Autofluorescence of renal tubuli is displayed in green; the NGAL or A1M staining signal is displayed in orange. Renal IRI and vehicle treatment only induced minor tubular NGAL staining (**A**,**E**). IRI + CFH resulted in significant increase in tubular NGAL staining (**C**,**E**). Tubular transport function was only slightly impaired in renal IRI and vehicle treatment as indicated by A1M tubular cast formation (**B**,**F**). However, CFH administration in mild renal IRI caused severe impairment of tubular transport function as evidenced by significantly increased A1M cast staining (**D**,**F**). ** *p* < 0.01, *** *p* < 0.001. Scale bar 100 µm.

Tubular neutrophil gelatinase-associated lipocalin (NGAL) staining was significantly higher in IRI + CFH mice than in IRI + vehicle mice (Figure 3A,C,E), whereas sham-operated and IRI-vehicle mice showed only minor tubular NGAL staining (Figure 3A,E). α1-microglobulin (A1M) tubular cast formation is a sign of impaired tubular transport function [16] and was significantly enhanced in IRI mice challenged with CFH compared with vehicle mice (Figure 3B,D,F).

**Figure 4:**
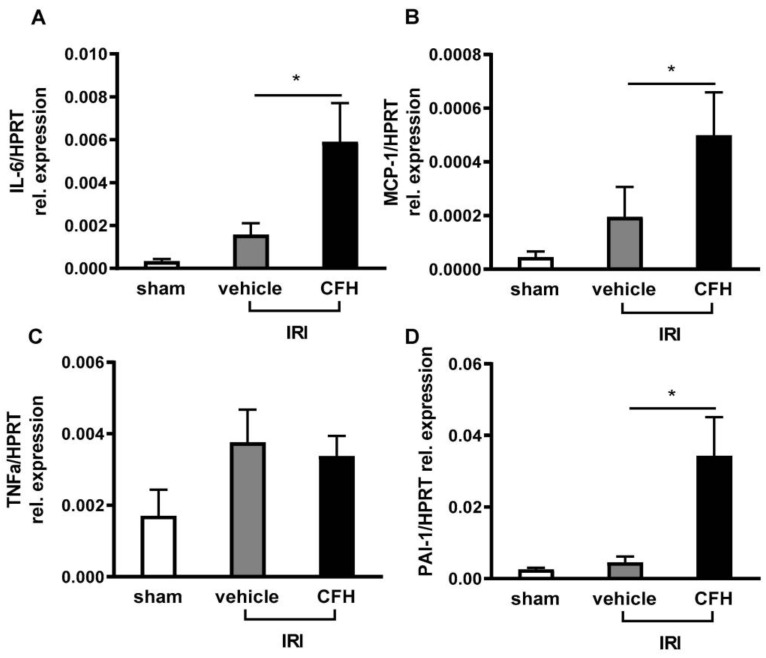
mRNA expressions of the proinflammatory cytokines IL-6 (**A**), MCP-1 (**B**), TNFα (**C**), and the profibrotic cytokine PAI-1 (**D**) in mice kidney tissue were analyzed 48 h after sham (white bar), IRI and vehicle (grey bar) and IRI + CFH administration (black bar). Renal RI with an ischemia time of 15 min did not cause significant renal upregulation of proinflammatory or profibrotic cytokines. In contrast, mice exposed to CFH after IRI exhibited enhanced renal expression of IL-6, MCP-1, and PAI-1. * *p* < 0.05.

Proinflammatory and profibrotic cytokine expressions in the kidney tissue were measured by quantitative PCR (qPCR). CFH administered in mild renal IRI mice caused marked renal upregulation of the proinflammatory cytokines interleukin (IL)-6 (Figure 4A), monocyte chemoattractant protein (MCP)-1 (Figure 4B), and tumor necrosis factor (TNF)α (Figure 4C), as well as the profibrotic cytokine plasminogen activator inhibitor (PAI)-1 (Figure 4D). The levels of these cytokines were not significantly elevated in sham surgery or subclinical renal IRI + vehicle treatment mice.

**Figure 5:**
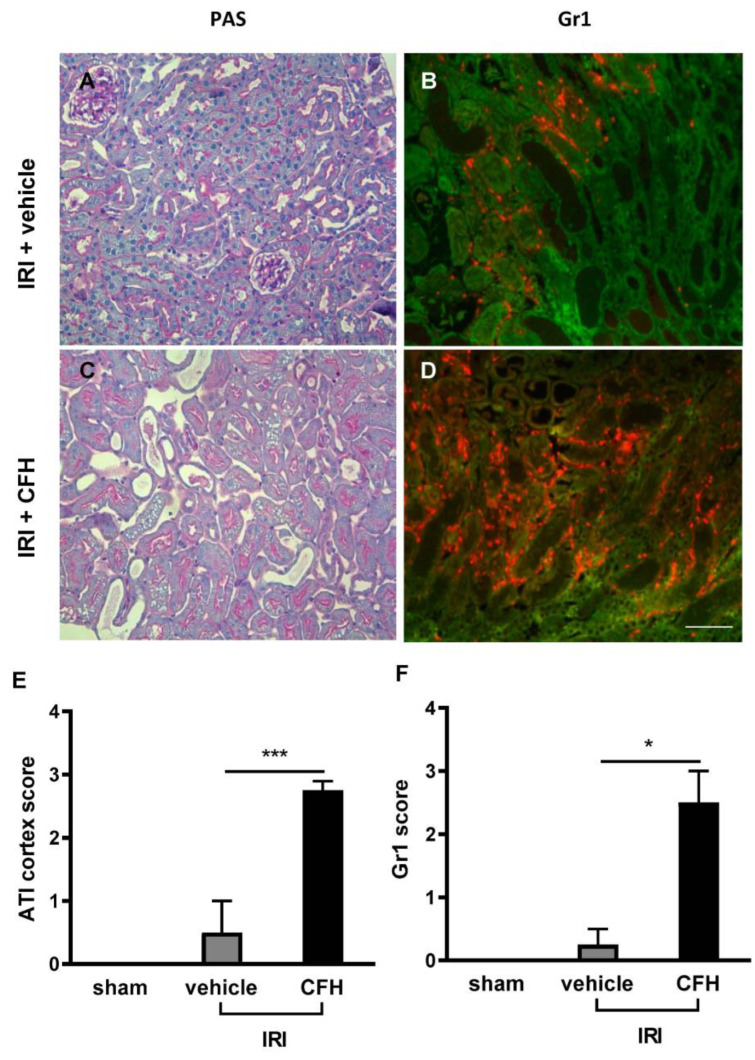
Renal morphology (PAS staining, (**A**,**C**)) and neutrophil infiltration (immunofluorescence for Gr-1 (**B**,**D**)) in mice 48 h after IRI with and without CFH treatment. The renal IRI model used in this study was characterized by only minor morphological kidney damage (**A**,**E**). However, CFH administration in renal IRI mice caused severe acute tubular injury (ATI) with loss of brush border, tubular dilatation, and tubular cast formation (**C**,**E**). After renal IRI and vehicle treatment, minor renal neutrophil infiltration was observed (**B**,**F**). In contrast, severe neutrophil infiltration was present 48 h after IRI and CFH treatment (**D**,**F**). * *p* < 0.05, *** *p* < 0.001. Scale bar 100 µm.

Mice exposed to mild renal IRI + vehicle displayed only minor histological damage (Figure 5A,E), whereas acute tubular injury (ATI) was significantly pronounced in the renal cortex of mice exposed to IRI + CFH (Figure 5C,E). Severe neutrophil infiltration in the outer medulla of the ischemic kidneys was present after CFH administration in renal IRI mice (Figure 5D,F).

In summary, exposure to CFH in this mouse model of transient mild (15 min) bilateral renal IRI caused overt AKI with kidney tissue damage and proinflammatory alterations.

## 3. Discussion

AKI occurs in 39–69% of patients after LuTx [21,22,23,24,25], depending on the criteria for defining AKI [26], and is associated with increased mortality [5,6]. Multiple factors can be relevant for the development of AKI after LuTx. Previous studies have shown that diabetic patients [27] and patients with supratherapeutic levels of tacrolimus [28] are at increased AKI risk following LuTx. In addition, severe arterial hypotension, intraoperative ECMO, and red blood cell transfusion have also been described as risk factors for AKI in the setting of LuTx [1,29,30,31].

So far, an association of increased CFH levels in LuTx patients and primary graft dysfunction has been demonstrated [20], but the role of CFH in AKI following LuTx is unknown. Transfusion of RBC units and shear-stress-induced hemolysis from ECMO are common in LuTx and the two major sources of CFH in this setting. The rationale for the current study was to determine the association of CFH with AKI in LuTx patients and to investigate whether exogenous CFH administration causes AKI when combined with a second injury factor relevant for LuTx-associated AKI: renal IRI.

In the clinical part of the study, we found that the CFH level was significantly increased and the haptoglobin level significantly decreased at the end of LuTx surgery. Furthermore, the levels of CFH correlated with the duration of surgery and serum creatinine levels. Despite the small sample size, the only clinical factors that were different between AKI and no-AKI patients in this LuTx cohort were the duration of surgery and lung ischemia, which may be associated with a complicated course of surgery. During tissue ischemia, free heme can be released into the circulation, which is known to be nephrotoxic [32] and might be an additional factor contributing to AKI development. In contrast with previous reports [30], the amount of transfused RBC units was comparable in patients with and without AKI in the current study. This might be contradictory at first glance, because higher CFH levels were observed in AKI patients and transfusion of packed RBCs is a major source of CFH. However, it was shown that the CFH levels in RBC units increase during prolonged storage periods [33]. Although transfusion of long-term stored RBC units did not affect mortality in critically ill patients [34], it was associated with increased AKI risk after liver transplantation [35]. Therefore, the increased CFH levels observed in LuTx patients with AKI might be due to the transfusion of aged RBC units. Moreover, few patients required intraoperative ECMO support, and there were no differences between the AKI and no-AKI groups (Table 1). The rest of the patients underwent surgery without extracorporeal circuit. Therefore, the pronounced CFH level increase in LuTx patients with AKI might be associated with the transfusion of stored RBCs.

In the experimental part of the study, the exogenous administration of CFH was found to promote overt AKI in subclinical renal IRI with marked serum creatinine elevation and ATI. Moreover, we found that CFH caused tubular A1M cast formation, indicating impairment of tubular transport function [36]. CFH induced a strong proinflammatory milieu with enhanced the renal expression of the proinflammatory cytokines IL-6, MCP-1, and TNFα and the severe kidney neutrophil infiltration that is usually observed after extended renal ischemia times of 35 min [37,38]. These findings suggest that CFH is a functional second hit that exacerbates renal IRI to overt AKI. Similar to our present findings, administration of CFH was previously shown to cause severe AKI with tubular injury in septic mice [16].

The current study has two limitations. First, the age of transfused RBC units was not recorded. Future studies will address whether CFH level elevation in the plasma of LuTx patients may correlate with the age of transfused RBC units. Second, mouse experiments lacked a sham control group treated with CFH because Shaver et al. previously showed that CFH administration to sham-operated mice did not cause renal injury in a mouse model on CFH and sepsis [16].

In summary, our current study shows that increased levels of CFH correlate with AKI in LuTx patients. The factors associated with increased CFH are prolonged lung ischemia and duration of surgery. The increased levels of CFH inversely correlated to haptoglobin depletion in LuTx patients with AKI. Experimentally, the exogenous CFH administered in subclinical renal IRI appears to cause a second hit that mediates AKI via tubular injury, renal inflammation, and tubular transport impairment.

In conclusion, we show that CFH may have an important role in the development of AKI after LuTx and is a causal injury factor in renal IRI. Targeting of CFH (e.g., with the scavenger protein haptoglobin) may serve as a therapeutic approach for counteracting its clinical toxicity in LuTx.

## 4. Methods and Materials

### 4.1. Clinical Study

#### 4.1.1. LuTx Patients

In a cohort of 185 adult double lung transplant patients prospectively enrolled into a clinical study at Hannover Medical School, Germany, during 2013 and 2014, *n* = 10 patients with and without AKI each were randomly selected for this study to measure CFH and haptoglobin. The study was approved by the local ethics committee (no. 6895), and written informed consent was obtained from all patients. Patient characteristics are summarized in Table 1. Surgical management of LuTx patients at our institution has been described previously [39,40]. LuTx surgery is usually performed without extracorporeal circulation such as ECMO. ECMO during surgery is applied in conditions associated with severe pulmonary hypertension or refractory hemodynamic instability [40]. All patients received initial triple immunosuppressive therapy by tacrolimus, mycophenolate mofetil, and steroids. Postoperative aggressive weaning from mechanical ventilation whenever possible was intended. The time points for sample collection were immediately recorded after anesthesia induction (baseline), at the end of surgery (surg.-end), and on day 1 after surgery (d1).

#### 4.1.2. Sample Preparation and Measurement of CFH and Haptoglobin

Following collection of blood from the patients’ central venous catheter, patient plasma was obtained by centrifugation at 200× *g* for 10 min and the supernatant was transferred to Eppendorf tubes and centrifuged again at 200× *g* for 10 min to remove residual RBCs. The supernatants obtained were aliquoted and immediately stored at −80 °C until further analysis. CFH (LSBio, Seattle, WA, USA) and haptoglobin (Abcam, Cambridge, UK) were measured using a commercially available ELISA kit in accordance with the descriptions from the manufacturer.

#### 4.1.3. AKI Definition

AKI was graded according to the Kidney Disease Improving Global Outcomes (KDIGO) criteria [41] into stage I–III based on serum creatinine (sCr) elevation during 48 h postsurgery as follows: stage I: sCr increase by 1.5- to 1.9-fold from baseline or absolute sCr increase by ≥0.3 mg/dL (≥26.5 μmol/L); stage II: sCr increase by 2.0- to 2.9-fold from baseline; stage III: sCr increase by 3.0-fold from baseline or absolute sCr increase by ≥4.0 mg/dL (≥353.6 μmol/L) or initiation of renal replacement therapy.

### 4.2. Experimental Study

#### 4.2.1. Animals

Male CD1 mice (7 weeks of age, body weight 30–35 g) purchased from Charles River (Sulzfeld, Germany) and housed at the Institute of Laboratory Animal Sciences, Hannover Medical School (Hannover, Germany) were used for the experiments. All mice had free access to drinking water and food and were kept under a day/night cycle of 14/10 h. All experiments were approved by the local animal protection committee of the Lower Saxony State department for animal welfare and food protection (33.19-42502-04-14/1657, Germany). Mice were monitored daily for physical condition after surgery.

#### 4.2.2. Renal Ischemia Reperfusion Injury (IRI)

Isoflurane was used for anesthesia (3% induction, 1–2% maintenance) and butorphanol (1 mg/kg) for analgesia. IRI was induced by bilateral renal pedicle clamping with a micro aneurysm clip (Aesculap, Tuttlingen, Germany) for 15 min, and reperfusion was visually assessed. Sham surgery with opening of the abdominal cavity for 15 min, but without manipulation of the renal vessels served as control. CFH (isolated from human packed red blood cells) was intravenously administered at a dose of 2.5 mg/mouse immediately after reperfusion. The vehicle group received injection of CFH buffer (20 mM Tris HCl, 100 mM NaCl, 0.1 mM CaCl_2_, pH 8.0, 5 µL/g). N = 5 mice from each group were used for experiments.

#### 4.2.3. Organ Preservation

Mice were sacrificed in deep general anesthesia (5% isoflurane) at 48 h after IRI, and organ retrieval was performed. After midline laparotomy, whole-body perfusion with ice-cold 0.9% PBS via the left cannulated ventricle resulted in circulatory arrest. Organs were dissected and fixed in RNA and later in 4% paraformaldehyde or shock-frozen in liquid nitrogen.

#### 4.2.4. Measurement of Serum Creatinine, BUN and Liver Enzymes

Serum creatinine, BUN, ALT, and AST levels were measured using an Olympus AU400 Chemistry Analyzer (Backman Coulter, California, CA, USA).

#### 4.2.5. Cytokine Expression

Total mRNA was isolated from cross-sectioned kidney slices using an RNeasy Mini Kit (Qiagen, Hilden, Germany), and cDNA was subsequently synthetized with Prime Script Reverse Transcriptase reagent (Takara, Japan) from DNase-treated total RNA. A LightCycler 96 (Roche, Penzberg, Germany) was used to conduct qPCR. The following primers were used: interleukin-6 (IL-6, Qiagen, #QT00098875), monocyte chemoattractant protein-1 (MCP-1, Qiagen, #QT00167832), tumor necrosis factor alpha (TNFα, Qiagen, #QT00104006), and plasminogen activator inhibitor 1 (PAI-1, BioTez, Berlin, Fwd:5′-ATGTTTAGTGCAACCCTGGC-3′, Rev: 5′-CTGCTCTTGGTCGGAAAGAC-3′). Hypoxanthine phosphoribosyl transferase (HPRT, Qiagen, #QT00166768) served as housekeeper for normalization.

#### 4.2.6. Renal Morphology and Immunofluorescence

After paraffin embedding, 2 µm sections were cut and stained with periodic acid Schiff (PAS) according to standard protocols. Acute tubular injury (ATI) scoring was conducted as previously described [36] in the renal cortex using a semiquantitative grading system: 0 = focal ATI with <5% of tubuli of the cortex affected, 1 = mild ATI with 5–25% of tubuli affected, 2 = moderate ATI with 26–50% of tubuli affected, 3 = severe ATI with 51–75% of the tubuli affected, and 4 = very severe ATI with >75% of tubuli affected. Immunofluorescence was performed with the following antibodies: Gr-1+ for neutrophils (Ly-6G/Ly-6C+, Serotec, UK), NGAL (Dianova, Biozol, Germany) and A1M (polyclonal rabbit anti-mouse A1M “Sven”, Lund, Sweden). Tubulointerstitial neutrophil infiltration in the outer medulla was semiquantitatively scored using the following score 0: <5 cells/view field (VF), 1: 5–10 cells/VF, 2: 11–20 cells/VF, 3: 21–50 cells/VF, and 4: >50 cells/VF, as previously described [37]. Tubular NGAL staining was semiquantitatively assessed using the following scoring system: 0 = < 5% of tubuli displaying NGAL staining, 1 = NGAL staining in 5–25% of tubuli, 2 = NGAL staining in 26–50% of tubuli, 3 = NGAL staining in 51–75% of the tubuli, and 4 = NGAL staining in >75% of tubuli. A1M tubular cast formation was quantified as percentage of the affected tubuli in 10 different areas. Analysis was conducted on a Leica imaging microscope (Leica, Wetzlar, Germany) at 200-fold magnification. Investigators were blinded to the group assignment. Images were captured with the same magnification.

#### 4.2.7. Statistical Analysis

Statistical analysis of the CFH and haptoglobin levels in the clinical study and the results of the experimental study was performed with GraphPad Prism, version 5.0 (GraphPad Software, San Diego, CA, USA). IBM SPSS Statistics, version 26 (SPSS Inc., IBM Corporation, Chicago, IL, USA) was used to determine the Spearman correlation of CFH with duration of surgery and s-creatinine level after 48 h and to perform statistical analysis of clinical patient data. The Mann–Whitney test was used to compare numerical data and Fisher’s exact test and the Chi-square test to compare categorical data. Multiple comparisons were analyzed by one-way ANOVA or Kruskal–Wallis, and group means were compared using Tukey’s post hoc test. Experimental data are reported as mean value ± standard error of the mean (SEM). *p*-values < 0.05 were accepted as significant.

## Figures and Tables

**Table 1:** Pre-, peri-, and postoperative data of LuTx patients (mean ± standard deviation).

	All pts. *n* = 20	No AKI *n* = 10	AKI *n* = 10	*p*-Value
Patients				
Age (years)	49.3 ± 11	48.7 ± 13	49.9 ± 8	0.813
Sex *n* (%; male/female)	10 (50)/10 (50)	4 (40)/6 (60)	6 (60)/4 (40)	0.371
Body mass index (kg/m^2^)	22.0 ± 3.9	21.1 ± 4.2	22.9 ± 3.5	0.773
Indication for transplantation. *n* (%)				
COPD/emphysema	10 (50)	5 (50)	5 (50)	-
Cystic fibrosis	2 (10)	1 (10)	1 (10)	-
Fibrosis	6 (30)	2 (20)	4 (40)	-
Other	2 (10)	2 (20)	0 (0)	-
Comorbidities				
Arterial hypertension. *n* (%)	5 (25)	3 (30)	2 (20)	0.606
Diabetes mellitus. *n* (%)	5 (25)	2 (20)	3 (30)	0.606
Preoperative factors				
FEV 1 (%)	25.0 ± 17	26.1 ± 20	23.8 ± 15	0.773
FVC (%)	40.6 ± 18	42.8 ± 19	38.4 ± 18	0.599
Baseline serum creatinine (µmol/L)	61.0 ± 15	63.8 ± 14	58.1 ± 16	0.396
Baseline eGFR (mL/min)	106 ± 15	103 ± 20	109 ± 10	0.400
Baseline serum C-reactive protein (mg/L)	11.2 ± 13	16.4 ± 17	6.0 ± 6	0.090
ECMO pre-operative. *n* (%)	1 (5)	0 (0)	1 (10)	0.305
Perioperative factors				
Duration of surgery (min)	302.5 ± 63	273.7 ± 44	331.2 ± 69	0.039
Ischemia time first side (min)	401.8 ± 98	356.0 ± 68	447.6 ± 105	0.033
Ischemia time second side (min)	518.8 ± 107	464.0 ± 76	573.5 ± 108	0.017
ECMO intraoperative. *n* (%)	5 (25)	2 (20)	3 (30)	0.606
Transfused packed red blood cells (pRBCs) during surgery	1.4 ± 1.8	1.2 ± 1.5	1.6 ± 2.0	0.624
Transfused fresh frozen plasma (FFP) during surgery	1.5 ± 1.9	1.9 ± 1.9	1.1 ± 1.9	0.380
Transfused platelets during surgery	0.3 ± 0.7	0.4 ± 0.9	0.2 ± 0.6	0.493
Serum creatinine 24 h (µmol/L)	92.8 ± 45	69.2 ± 18	116.4 ± 52	0.019
Serum creatinine 48 h (µmol/L)	87.9 ± 48	61.8 ± 18	113.9 ± 55	0.016
eGFR 24 h (mL/min)	82 ± 30	97 ± 24	66 ± 29	0.019
eGFR 48 h (mL/min)	87 ± 36	104 ± 25	73 ± 34	0.026
Reoperation. *n* (%)	4 (20)	1 (10)	3 (30)	0.264
Transfused packed red blood cells (pRBC) postoperative	2.5 ± 2.0	2.5 ± 2.4	2.5 ± 1.8	1.000
ECMO postoperative. *n* (%)	2 (10)	1 (10)	1 (10)	1.000
Hospital stay (days)	28.2 ± 12	24.2 ± 6	32.1 ± 15	0.149
Intensive care unit treatment (days)	3.8 ± 4	2.8 ± 2	4.7 ± 4	0.257

Abbreviations: COPD, chronic obstructive pulmonary disease; ECMO, extracorporeal membrane oxygenation; eGFR, estimated glomerular filtration rate; FEV 1, forced expiratory volume in 1 s; FVC, forced vital capacity; h, hours; pRBCs, packed red blood cells; Tx, transplantation.

## Data Availability

Original data are available upon request from the corresponding author.

## References

[1] Bennett D., Fossi A., Marchetti L., Lanzarone N., Sisi S., Refini R.M., Sestini P., Luzzi L., Paladini P., Rottoli P. (2019). Postoperative acute kidney injury in lung transplant recipients. Interact. Cardiovasc. Thorac. Surg..

[2] Balci M.K., Vayvada M., Salturk C., Kutlu C.A., Ari E. (2017). Incidence of Early Acute Kidney Injury in Lung Transplant Patients: A Single-Center Experience. Transpl. Proc..

[3] Wehbe E., Duncan A.E., Dar G., Budev M., Stephany B. (2013). Recovery from AKI and short- and long-term outcomes after lung transplantation. Clin. J. Am. Soc. Nephrol..

[4] Karkouti K., Wijeysundera D.N., Yau T.M., Callum J.L., Cheng D.C., Crowther M., Dupuis J.Y., Fremes S.E., Kent B., Laflamme C. (2009). Acute kidney injury after cardiac surgery: Focus on modifiable risk factors. Circulation.

[5] Hobson C.E., Yavas S., Segal M.S., Schold J.D., Tribble C.G., Layon A.J., Bihorac A. (2009). Acute kidney injury is associated with increased long-term mortality after cardiothoracic surgery. Circulation.

[6] Atchade E., Barour S., Tran-Dinh A., Jean-Baptiste S., Tanaka S., Tashk P., Snauwaert A., Lortat-Jacob B., Mourin G., Mordant P. (2020). Acute Kidney Injury After Lung Transplantation: Perioperative Risk Factors and Outcome. Transpl. Proc..

[7] Jing L., Chen W., Zhao L., Guo L., Liang C., Chen J., Wang C. (2021). Acute kidney injury following adult lung transplantation. Chin. Med. J..

[8] Kim N.E., Kim C.Y., Kim S.Y., Kim H.E., Lee J.G., Paik H.C., Park M.S. (2021). Risk factors and mortality of acute kidney injury within 1 month after lung transplantation. Sci. Rep..

[9] Reeder B.J. (2010). The redox activity of hemoglobins: From physiologic functions to pathologic mechanisms. Antioxid. Redox Signal..

[10] Schaer D.J., Buehler P.W., Alayash A.I., Belcher J.D., Vercellotti G.M. (2013). Hemolysis and free hemoglobin revisited: Exploring hemoglobin and hemin scavengers as a novel class of therapeutic proteins. Blood.

[11] Van Avondt K., Nur E., Zeerleder S. (2019). Mechanisms of haemolysis-induced kidney injury. Nat. Rev. Nephrol..

[12] Fan X., Zhang X., Liu L.C., Kim A.Y., Curley S.P., Chen X., Dworkin L.D., Cooper C.J., Gupta R. (2022). Interleukin-10 attenuates renal injury after myocardial infarction in diabetes. J. Investig. Med..

[13] Moussavian M.R., Slotta J.E., Kollmar O., Menger M.D., Schilling M.K., Gronow G. (2007). Hemoglobin induces cytotoxic damage of glycine-preserved renal tubules. Transpl. Int..

[14] Baek J.H., D’Agnillo F., Vallelian F., Pereira C.P., Williams M.C., Jia Y., Schaer D.J., Buehler P.W. (2012). Hemoglobin-driven pathophysiology is an in vivo consequence of the red blood cell storage lesion that can be attenuated in guinea pigs by haptoglobin therapy. J. Clin. Investig..

[15] Chintagari N.R., Nguyen J., Belcher J.D., Vercellotti G.M., Alayash A.I. (2015). Haptoglobin attenuates hemoglobin-induced heme oxygenase-1 in renal proximal tubule cells and kidneys of a mouse model of sickle cell disease. Blood Cells Mol. Dis..

[16] Shaver C.M., Paul M.G., Putz N.D., Landstreet S.R., Kuck J.L., Scarfe L., Skrypnyk N., Yang H., Harrison F.E., de Caestecker M.P. (2019). Cell-free hemoglobin augments acute kidney injury during experimental sepsis. Am. J. Physiol. Renal. Physiol..

[17] Adamzik M., Hamburger T., Petrat F., Peters J., de Groot H., Hartmann M. (2012). Free hemoglobin concentration in severe sepsis: Methods of measurement and prediction of outcome. Crit. Care.

[18] Omar H.R., Mirsaeidi M., Socias S., Sprenker C., Caldeira C., Camporesi E.M., Mangar D. (2015). Plasma Free Hemoglobin Is an Independent Predictor of Mortality among Patients on Extracorporeal Membrane Oxygenation Support. PLoS ONE.

[19] Graw J.A., Hildebrandt P., Krannich A., Balzer F., Spies C., Francis R.C., Kuebler W.M., Weber-Carstens S., Menk M., Hunsicker O. (2022). The role of cell-free hemoglobin and haptoglobin in acute kidney injury in critically ill adults with ARDS and therapy with VV ECMO. Crit. Care.

[20] Shaver C.M., Wickersham N., McNeil J.B., Nagata H., Miller A., Landstreet S.R., Kuck J.L., Diamond J.M., Lederer D.J., Kawut S.M. (2018). Cell-free hemoglobin promotes primary graft dysfunction through oxidative lung endothelial injury. JCI Insight.

[21] Rocha P.N., Rocha A.T., Palmer S.M., Davis R.D., Smith S.R. (2005). Acute renal failure after lung transplantation: Incidence, predictors and impact on perioperative morbidity and mortality. Am. J. Transplant..

[22] Arnaoutakis G.J., George T.J., Robinson C.W., Gibbs K.W., Orens J.B., Merlo C.A., Shah A.S. (2011). Severe acute kidney injury according to the RIFLE (risk, injury, failure, loss, end stage) criteria affects mortality in lung transplantation. J. Heart Lung Transplant..

[23] Jacques F., El-Hamamsy I., Fortier A., Maltais S., Perrault L.P., Liberman M., Noiseux N., Ferraro P. (2012). Acute renal failure following lung transplantation: Risk factors, mortality, and long-term consequences. Eur. J. Cardiothorac. Surg..

[24] Wehbe E., Brock R., Budev M., Xu M., Demirjian S., Schreiber M.J., Stephany B. (2012). Short-term and long-term outcomes of acute kidney injury after lung transplantation. J. Heart Lung Transplant..

[25] Fidalgo P., Ahmed M., Meyer S.R., Lien D., Weinkauf J., Cardoso F.S., Jackson K., Bagshaw S.M. (2014). Incidence and outcomes of acute kidney injury following orthotopic lung transplantation: A population-based cohort study. Nephrol. Dial. Transplant..

[26] Lassnigg A., Schmidlin D., Mouhieddine M., Bachmann L.M., Druml W., Bauer P., Hiesmayr M. (2004). Minimal changes of serum creatinine predict prognosis in patients after cardiothoracic surgery: A prospective cohort study. J. Am. Soc. Nephrol..

[27] Wajda-Pokrontka M., Nadziakiewicz P., Krauchuk A., Ochman M., Zawadzki F., Przybylowski P. (2022). Incidence and Perioperative Risk Factors of Acute Kidney Injury among Lung Transplant Recipients. Transplant. Proc..

[28] Sikma M.A., Hunault C.C., van de Graaf E.A., Verhaar M.C., Kesecioglu J., de Lange D.W., Meulenbelt J. (2017). High tacrolimus blood concentrations early after lung transplantation and the risk of kidney injury. Eur. J. Clin. Pharmacol..

[29] Doricic J., Greite R., Vijayan V., Immenschuh S., Leffler A., Ius F., Haverich A., Gottlieb J., Haller H., Scheffner I. (2022). Kidney injury after lung transplantation: Long-term mortality predicted by post-operative day-7 serum creatinine and few clinical factors. PLoS ONE.

[30] Botros M., Jackson K., Singh P., Rosenheck J.P., Ganapathi A.M., Henn M.C., Howsare M.M., Mokadam N.A., Pesavento T., Whitson B.A. (2022). Insights into early postoperative acute kidney injury following lung transplantation. Clin. Transplant..

[31] Knight J., Hill A., Melnyk V., Doney L., D’Cunha J., Kenkre T., Subramaniam K., Howard-Quijano K. (2022). Intraoperative Hypoxia Independently Associated With the Development of Acute Kidney Injury Following Bilateral Orthotopic Lung Transplantation. Transplantation.

[32] Wang L., Vijayan V., Jang M.S., Thorenz A., Greite R., Rong S., Chen R., Shushakova N., Tudorache I., Derlin K. (2019). Labile Heme Aggravates Renal Inflammation and Complement Activation After Ischemia Reperfusion Injury. Front. Immunol..

[33] Donadee C., Raat N.J., Kanias T., Tejero J., Lee J.S., Kelley E.E., Zhao X., Liu C., Reynolds H., Azarov I. (2011). Nitric oxide scavenging by red blood cell microparticles and cell-free hemoglobin as a mechanism for the red cell storage lesion. Circulation.

[34] Cooper D.J., McQuilten Z.K., Nichol A., Ady B., Aubron C., Bailey M., Bellomo R., Gantner D., Irving D.O., Kaukonen K.M. (2017). Age of Red Cells for Transfusion and Outcomes in Critically Ill Adults. N. Engl. J. Med..

[35] Wang Y., Li Q., Ma T., Liu X., Wang B., Wu Z., Dang S., Lv Y., Wu R. (2018). Transfusion of Older Red Blood Cells Increases the Risk of Acute Kidney Injury After Orthotopic Liver Transplantation: A Propensity Score Analysis. Anesth. Analg..

[36] Rund K.M., Peng S., Greite R., Claassen C., Nolte F., Oger C., Galano J.M., Balas L., Durand T., Chen R. (2020). Dietary omega-3 PUFA improved tubular function after ischemia induced acute kidney injury in mice but did not attenuate impairment of renal function. Prostaglandins Other Lipid Mediat..

[37] Greite R., Thorenz A., Chen R., Jang M.S., Rong S., Brownstein M.J., Tewes S., Wang L., Baniassad B., Kirsch T. (2018). Renal ischemia-reperfusion injury causes hypertension and renal perfusion impairment in the CD1 mice which promotes progressive renal fibrosis. Am. J. Physiol. Renal. Physiol..

[38] Greite R., Derlin K., Hensen B., Thorenz A., Rong S., Chen R., Hellms S., Jang M.S., Brasen J.H., Meier M. (2020). Early antihypertensive treatment and ischemia-induced acute kidney injury. Am. J. Physiol. Renal. Physiol..

[39] Draeger H., Salman J., Aburahma K., Becker L.S., Siemeni T., Boethig D., Sommer W., Avsar M., Bobylev D., Schwerk N. (2021). Impact of unilateral diaphragm elevation on postoperative outcomes in bilateral lung transplantation—A retrospective single-center study. Transpl. Int..

[40] Ius F., Sommer W., Tudorache I., Avsar M., Siemeni T., Salman J., Molitoris U., Gras C., Juettner B., Puntigam J. (2016). Five-year experience with intraoperative extracorporeal membrane oxygenation in lung transplantation: Indications and midterm results. J. Heart Lung Transplant..

[41] Okusa M.D., Davenport A. (2014). Reading between the (guide)lines—The KDIGO practice guideline on acute kidney injury in the individual patient. Kidney Int..

